# An Intervention to Optimize Coach Motivational Climates and Reduce Athlete Willingness to Dope (CoachMADE): Protocol for a Cross-Cultural Cluster Randomized Control Trial

**DOI:** 10.3389/fpsyg.2017.02301

**Published:** 2018-01-09

**Authors:** Nikos Ntoumanis, Daniel F. Gucciardi, Susan H. Backhouse, Vassilis Barkoukis, Eleanor Quested, Laurie Patterson, Brendan J. Smith, Lisa Whitaker, George Pavlidis, Stela Kaffe

**Affiliations:** ^1^School of Psychology, Curtin University, Bentley, WA, Australia; ^2^School of Physiotherapy and Exercise Science, Curtin University, Perth, WA, Australia; ^3^Institute for Sport Physical Activity and Leisure, Leeds Beckett University, Leeds, United Kingdom; ^4^Department of Physical Education and Sport Sciences, Aristotle University of Thessaloniki, Thessaloniki, Greece

**Keywords:** anti-doping, motivation, coach training, self-determination theory

## Abstract

Field-based anti-doping interventions in sport are scarce and focus on athletes. However, coaches are recognized as one of the most significant source of influence in terms of athletes’ cognitions, affect, and behavior. In this paper, we present the protocol for a cluster randomized control trial which aims to contrast the relative effects of a ‘motivation and anti-doping’ intervention program for coaches against an information-based anti-doping control program. In developing the motivation content of our intervention, we drew from Self-Determination Theory. The project is currently ongoing in Australia and has recently started in the United Kingdom and Greece. We aim to recruit 120 coaches and approximately 1200 of their athletes across the three countries. Various assessments will be taken from both coaches and athletes prior to the intervention, immediately after the 12-week intervention and at a 2-month follow up. The intervention comprises face-to-face workshops and weekly activities which are supported by printed and online material. The project aims to identify communication strategies that coaches can use to support athletes’ motivation in sport and also to promote self-determined reasons for athletes to comply with doping regulations.

**Trial Registration:** The trial is registered with the Australia and New Zealand Clinical Trials Registry (ANZCTR): ACTRN12616001688471.

## Introduction

Despite past and current anti-doping efforts by many national and international anti-doping agencies, instances of doping in sport continue to capture media headlines worldwide. For example, in August 2015, the Sunday Times alleged that data from 5,000 Track and Field athletes revealed an “extraordinary extent of cheating” (Calvert et al., 2015). Doping contravenes the fundamental principles of Olympism and the Olympic charter and it can also harm athletes’ health (International Olympic Committee, 2014). As such, the “zero tolerance” response to doping allegations by the International Olympic Committee is unsurprising. However, in addition to a strong punitive stance, anti-doping researchers (e.g., Barkoukis et al., 2013; Whitaker et al., 2014; Barkoukis, 2015) have argued for the merits of a preventative stance by fostering athletes’ anti-doping attitudes, diminished willingness to dope, and efficacy to resist doping-related temptations. Hence, our project aims to develop and test a preventative theory- and evidence-based intervention to reduce athletes’ willingness to take potentially banned substances via helping coaches to support athletes’ adaptive motivation and increasing coaches’ efficacy to discuss anti-doping information with their athletes in a motivationally supportive manner.

The first comprehensive meta-analysis of psycho-social predictors and outcomes of doping in sport by Ntoumanis et al. (2014) revealed two significant gaps in the anti-doping literature, both of which will be addressed via our project. First, the review showed the dearth of field-based intervention studies. An exception are the ATLAS and ATHENA programs which, however, offered a broad educational program which aimed to tackle other behaviors (e.g., drug use, healthy nutrition) in addition to doping (Goldberg et al., 1996; Elliot et al., 2004). These were athlete-centered interventions and were effective in reducing self-reported use of diet pills and body-shaping substances (e.g., anabolic steroids, and muscle-building supplements). The second research gap identified in the Ntoumanis et al. (2014) meta-analysis was that past literature has mainly centered on the role of personal variables (e.g., attitudes, beliefs, perfectionism) in predicting doping intentions and doping use. Research evidence on the role of socio-contextual factors is comparatively scarce and has focused primarily on the role of prevailing social norms in condoning or sanctioning doping behavior. Although this work is important in identifying the influence of prevailing social norms on doping-related variables, it does not capture the specific behaviors of others via which the social context exerts its influence on athletes. From a doping prevention perspective this exclusion is problematic; if researchers are to develop effective prevention programs, they need to be able to identify specific coach behaviors that should be fostered or avoided.

### Coach Communication Style

Although there are various influential social agents in sport (e.g., parents, peers, medical personnel, sport scientists), undoubtedly coaches play a crucial role in shaping the psychological experiences and actions of athletes (Bartholomew et al., 2009; Smith et al., 2010). Indeed, conceptual models of doping behavior (e.g., Donovan et al., 2002; Donahue et al., 2006; Johnson, 2012) and empirical evidence (e.g., Barkoukis et al., 2011; Bahrke, 2012) acknowledge the important role of the communication style used by coaches in predicting doping-related outcomes. Hence, it is surprising there are no published intervention studies in the doping literature that have trained coaches to promote an anti-doping environment by focusing on the motivational strategies adopted when they communicate with their athletes. Coaches instruct and try to motivate their athletes in ways in which they were coached themselves, or regard as most effective, or culturally acceptable or indicative of competent and authoritative instruction (Reeve, 2009). However, some of these motivational strategies are problematic and counterproductive.

Researchers in the sport motivation field have used Self-Determination Theory (SDT; Deci and Ryan, 2002), one of the most widely applied theories of motivation, to differentiate between adaptive and maladaptive coach strategies, and to investigate the effects of these strategies on athletes’ psychological needs, well-being, and behavior. In SDT research, a broad distinction has been made between need supportive and need thwarting motivational strategies (also called coach behaviors). Need supportive strategies aim to foster athletes’ three fundamental psychological needs: autonomy (feeling control over one’s own behavior), competence (feeling effective in producing desired outcomes), and relatedness (feeling connected with and accepted by others). Examples of need supportive behaviors include the provision of meaningful choice and rationale, taking others’ perspective into account, acknowledging their feelings, and providing feedback on competence that does not control others’ actions (Mahoney et al., 2017; Ntoumanis et al., 2018). Such behaviors can increase athletes’ psychological need satisfaction, well-being, and prosocial behavior (Mageau and Vallerand, 2003; Hodge and Lonsdale, 2011). In contrast, controlling behaviors are evident when coaches act in a coercive, pressuring, and authoritarian way in order to impose a specific and preconceived way of thinking and behaving upon their athletes. Need thwarting social environments can frustrate basic psychological needs and undermine psychological and physical wellness. For example, self-destructive behaviors (e.g., drug abuse) have been documented when individuals’ experience hostile social environments which thwart their needs (Deci and Ryan, 2000). Such findings have important implications for anti-doping research, as they highlight the role of social environments in affecting athletes’ welfare. However, there is no experimental research that has examined the role of contextual motivational factors (i.e., coaches’ need supportive and need thwarting behaviors) in predicting doping-related outcomes in athletes (e.g., attitudes to doping, willingness to take potentially illegal substances), via affecting athletes’ psychological need satisfaction and need frustration.

### Coach Communication Style and Athlete Doping

Ntoumanis et al. (2017a) utilized a prospective survey design to examine how coach communication style predicted doping-related variables among 166 Greek athletes. The findings indicated that continued self-reported doping use (at the beginning and the end of the sport season) was predicted indirectly and in a negative fashion by perceptions of coach autonomy (i.e., need) support via the moral attitude of “keeping winning in proportion.” Intentions to dope were also negatively predicted by need satisfaction via the same moral attitude. In contrast, perceptions of need thwarting coaching were positive indirect predictors of continued doping use via psychological need frustration, moral disengagement in doping (i.e., cognitively restructuring and discounting doping and its consequences), and endorsement of cheating. The authors argued that their findings could serve as a basis for developing anti-doping education programs for coaches with the aim of training them in more need supportive and less need thwarting behaviors. Our project aims to address this recommendation.

An important question for the potential usefulness of coach education is, do coaches engage in anti-doping education programs? Evidence suggests that coaches are reluctant to do so. Patterson et al. (2014b) presented evidence indicating low response rates from coaches in the United Kingdom and beyond to engage in such programs, due to perceived lack of personal relevance. Such reluctance is in stark contrast with the findings that emerged from the interviews of individuals responsible for anti-doping education in national and international sport and anti-doping organizations. In these interviews, carried out by the same authors (Patterson et al., 2014a), the administrators highlighted the importance of providing anti-doping education for coaches. In addition to logistical and resource challenges, the administrators identified negative perceptions of ‘anti-doping’ efforts (e.g., being punitive as opposed to informational) as an additional barrier to recruit coaches. In the same interviews, the administrators identified the need to obtain the ‘buy in’ from top administrators within a club or sport organization as a means of creating an appropriate ‘anti-doping culture’ within a club and engaging coaches to anti-doping education. In our project, we have followed this recommendation by engaging sport administrators and national or regional sport governing bodies.

In another interview study of Australian and Greek coaches, Ntoumanis et al. (2015) found that coaches had an aspiration to influence athletes’ doping-related decisions, but they lacked the efficacy or were unable to articulate the specific means by which they can facilitate the fight against doping. Besides feeling efficacious to deliver anti-doping education, it is important that coaches are upskilled to communicate such information in need supportive ways, and avoid or minimize a need thwarting interpersonal style. In the motivational literature, there has been a growing interest in delivering SDT-based interventions that aim to facilitate optimal motivational environments via need supportive communication styles among coaches, teachers, health professionals, and employers. A meta-analysis by Su and Reeve (2011) showed that such training programs were effective (weighted effect size *d* = 0.63).

The effects of such motivational interventions in terms of athlete doping-related attitudes, willingness to dope, and doping behavior have not yet been tested by any research team to date. In our project, we assess a number of outcomes of such an intervention at the athlete level. These outcomes are listed in italics in this and the next paragraph, alongside a brief justification or evidence of their relevance to doping research. Whitaker et al. (2014) application of the prototype/willingness model (Gibbons et al., 1998) for doping use in sport showed that *willingness to dope* was predicted by, amongst other things, *past doping behavior* and *pro-doping attitudes*. Similar findings with regard to the predictive role of the latter two variables were also reported by Barkoukis et al. (2013). In addition, Barkoukis and his colleagues found that *the efficacy to resist the doping-related temptations* was an important predictor of doping intentions and self-reported doping use. Further, as mentioned above, *moral disengagement in doping* has been identified as another strong predictor of doping intentions and doping use (Ntoumanis et al., 2017a).

Decisions to engage in doping are not always intentional. At times, athletes may risk the chance of inadvertent doping by taking an unknown substance, especially when they lack or have limited *anti-doping knowledge*. Morente-Sanchez and Zabala’s (2013) review identified that athletes lack anti-doping knowledge, particularly around dietary supplements and the possible side effects of performance enhancing drugs. Increasing such knowledge is one way to reduce both intentional and inadvertent doping use. In the same review, it was concluded that coaches were the main influence and source of information for athletes regarding anti-doping. Therefore, in addition to directly targeting athletes via educational programs and resources, improving coaches’ anti-doping education can also have indirect benefits in terms of athletes’ anti-doping knowledge. Yet Chan et al. (2016) also identified the importance of athletes improving their self-monitoring behaviors in order to avoid inadvertent doping. Hence, in this project we measure the number of *behaviors that athletes will adopt to prevent inadvertent doping* (e.g., checking medications for banned substances prior to use).

Besides collecting data from athletes, this project will also be the first intervention study in the anti-doping literature that will collect data from coaches. We assess coaches’ *use of need supportive and need thwarting communication style* when discussing doping related issues, as well as their *efficacy to discuss doping with athletes* and *create an anti-doping culture* within their team. Previous work on doping has used the term confrontation efficacy to refer to the efficacy of coaches to confront athletes about doping (Sullivan et al., 2015). However, we believe the term ‘confrontation’ is in contrast with the principles of motivational training in our project, hence, we have focused on situations in which the coach communicates and discusses doping with their athletes. To take this approach one step further, we ask coaches to rate their efficacy of initiating such discussions as well as the *perceived effectiveness of need supportive vs need thwarting style in dealing with a doping-related situation.* Similar to the athlete sample and for the same reasons as those given above, we also assess *coaches’ knowledge about anti-doping testing procedures* and *encouragement of their athletes to engage in inadvertent doping prevention behaviors.* Lastly, and again similar to the athlete sample, we measure coaches’ *anti-doping attitudes* and *moral disengagement in doping*. A study by Psouni et al. (2015) found that coaches’ intentions to encourage doping use amongst their athletes were strongly predicted by coaches’ pro-doping attitudes. Although there are no studies assessing coaches’ moral disengagement in doping, we suspect that this variable might also be linked to similar coach intentions.

### Objectives

The overarching aim of this project is to contrast the relative effects of an SDT-informed ‘motivation and anti-doping’ intervention program against a standard (i.e., information-based, increasing awareness) anti-doping control program. The intervention program focuses on developing need supportive communication strategies that coaches can apply when interacting with their athletes in general and specifically with regard to doping-related issues (e.g., checking for banned substances in medications). The standard anti-doping information program includes up-to-date information on various anti-doping issues (e.g., World Anti-Doping Agency’s Prohibited List, testing procedures, risk minimization process for using nutritional supplements), but excludes any motivation-related content.

We also aim to implement a process evaluation of the intervention via coach interviews, athlete interviews, coach questionnaires on ease and usefulness of the training material, as well as coach fidelity to the intervention material. We will disseminate the results of the intervention via coach information sessions, printed material, policy briefings, media interviews, social media engagement, and publications in peer reviewed journals.

### Hypotheses

• Compared to their baseline levels and to athletes in the control condition, athletes whose coaches complete the training will report: (1) less willingness to take potentially illegal substances (primary outcome), (2) higher perceptions of need supportive and lower perceptions of need thwarting coach motivational strategies (our manipulation check), (3) less favorable attitudes, (4) lower moral disengagement toward doping, (5) higher efficacy to resist doping-related temptations, (6) increased knowledge about anti-doping procedures, and (7) more behaviors to prevent unintentional/inadvertent doping. We also measure self-reported use of performance/recreational substances and drugs, but given that previous studies have found that only 10% of athletes admit to doping use (e.g., Barkoukis et al., 2013; Ntoumanis et al., 2017a), we do not expect to have statistical power to detect significant changes in such use.
• The intervention effects on doping-related variables will be mediated via increased psychological need satisfaction/reduced need frustration in the athletes in the experimental condition.
• Compared to their baseline levels and to coaches in the control condition, coaches who complete the intervention will (1) utilize more need supportive and less need thwarting communication strategies when discussing doping related issues with their athletes, (2) report higher efficacy to discuss doping with athletes and create an anti-doping atmosphere within the team, (3) rate need supportive communication styles as being more effective (need thwarting style as less effective) in dealing with a doping-related situations, (4) have better knowledge about anti-doping testing procedures, (5) encourage their athletes to use more inadvertent doping prevention behaviors, (6) report stronger anti-doping attitudes, and (7) report lower moral disengagement in doping.
• There is insufficient prior evidence to put forward hypotheses regarding any cross-cultural differences in the effectiveness of the intervention.

## Method

The reporting in this protocol paper follows the guidelines listed in the PRISMA and TIDieR checklists (see Supplementary Tables S1, S2). The project has three main phases, hence we present the participants and procedures in each phase separately.

### Phase 1

During Phase 1, we customized content from an existing theory- and evidence-based motivation intervention program to generate doping-specific content for the intervention condition. For comparison purposes, we also developed a “standard” anti-doping information program with no reference to motivational issues. All materials were translated in Greek and then translation was checked by the first and fourth authors who are fluent in English and Greek; minor modifications were made, where necessary, in the wording. In this phase, we also determined the assessment tools (i.e., questionnaires, role play scenarios, and workshop evaluation forms) to be used for Phase 2.

### Content Design

In total, three workshops were designed for the project. The intervention package comprised two 3-h workshops, whereas the control condition was one, 1-h workshop.

#### Control Workshop

The control workshop was designed by the United Kingdom-based team using traditional anti-doping information disseminated by National Anti-Doping Organizations (NADO; e.g., United Kingdom Anti-Doping; Australian Sport Anti-Doping Agency) and the World Anti-Doping Agency [WADA] (2015). A new workshop was designed because current national anti-doping education programs in the three countries are different in content and duration, hence, lacking the homogeneity needed for a ‘control’ comparison. Typically, anti-doping education involves the provision of information about anti-doping rules and regulations to ensure compliance and prevent an anti-doping rule violation. Topics that tend to be covered include the anti-doping rule violations, the prohibited substances list, therapeutic use exemptions, testing procedures, and checking the contents of supplements and medications. Often workshops of this kind occur as a one-off education session for coaches and athletes, typically lasting 60 min. The content provided in the control workshop has been designed to be consistent across the three countries. However, in places, presentation slides have been adapted so they are relevant to each country. For example, each country is directed to their own NADO to obtain further information (e.g., Australian Sports Anti-Doping Authority^1^). In addition, one of the tools to check medications is currently unavailable in Greece. This tool is a website known as Global Drug Reference Online (DRO). Global DRO does not currently cater for checking medications available in Greece, therefore participants in Greece are directed to the WADA Prohibited List and to the Gallinos website which provides advice on pharmaceutical products^2^.

#### Experimental Workshops

##### Workshop 1- motivation

Workshop 1 was designed by the Australian-based team. The focus of the first workshop is to provide education for coaches on how to implement a need supportive communication style with their athletes. Our aim is to help coaches understand how to build need supportive communication language in their general coaching, before trying to apply this skill to anti-doping discussions. This workshop and all associated resources are an adaption of an existing motivational workshop which has been previously delivered to fitness instructors in another trial (Hancox et al., 2015; Ntoumanis et al., 2017b).

The first workshop aims to achieve the following:

• Expand coaches’ understanding of motivation.
• Explore how/why subtleties in communication styles can be critical in supporting or undermining athletes’ motivation.
• Enhance coaches’ communication skills by applying motivational strategies based on contemporary research drawing primarily from SDT.
• Support coaches in practical ways (e.g., via brainstorming, role playing) to put into practice some of the taught material.
• Prepare coaches to implement a weekly program of motivation-related communication strategies and activities that they can apply to practice the skills learnt in the workshop over the next 4 weeks.

##### Workshop 2- motivation and anti-doping

Workshop 2 was designed by the Australian and United Kingdom teams. At the beginning of the second workshop, successes and pitfalls in implementing the taught material from the first workshop are discussed. In addition, reflective diaries are reviewed, and further advice is offered as coaches problem solve together to overcome obstacles in implementation. Following this activity, the main aims of Workshop 2 are to:

• Increase coaches’ knowledge of anti-doping rules and regulations and raise their awareness of the tools available to reduce the risk of inadvertent doping.
• Upskill coaches in how to apply need supportive communication, drawing from motivational principles outlined in Workshop 1, to reduce athletes’ willingness to dope and risk of inadvertent doping.

The material of Workshop 2 is specific to doping in terms of showing how motivational strategies can support or undermine doping-related variables at the athlete level. For example, coaches are trained to become more aware of how psychologically controlling tactics and pressuring techniques that focus on success at any costs can push athletes to use prohibited substances in an effort to appease their coach and gain their approval. It is also discussed how need thwarting coach environments can make athletes more susceptible to inadvertent doping because of increased adeptness at taking risks to gain coach approval. In contrast, it is shown how fostering athletes’ initiative, acknowledging their negative emotions, and offering unconditional support can prevent the development of contingencies in the athlete-coach relationship. Hence, under such circumstances athletes are less likely to resort to doping use as means of validating their self-worth and proving themselves to their coach, and are less susceptible to inadvertent doping. Such adaptive and maladaptive motivational strategies, and many other similar examples, are incorporated into our coach education program to help coaches self-reflect on their own motivational strategies and critically appraise how they influence their athletes’ willingness to take potentially illegal substances.

The training material helps coaches share existing anti-doping resources and communicate about doping with their athletes in a more need supportive manner and less need thwarting manner, using some of the aforementioned strategies. For example, emphasis is placed on providing rationales, acknowledging anxieties or uncertainties, being responsive to questions, taking time to listen to athletes’ opinions, and avoiding personal attacks, imposed goals, or intimidating tactics (see Ntoumanis et al., 2018). Given the influential role of peers on doping-decision making aspects (e.g., Woolf et al., 2014), coaches are trained to instigate such discussions both individually and in groups of athletes, so that athletes can also be educated as to how their communications and interactions with fellow athletes can influence doping-related outcomes.

**FIGURE 1:**
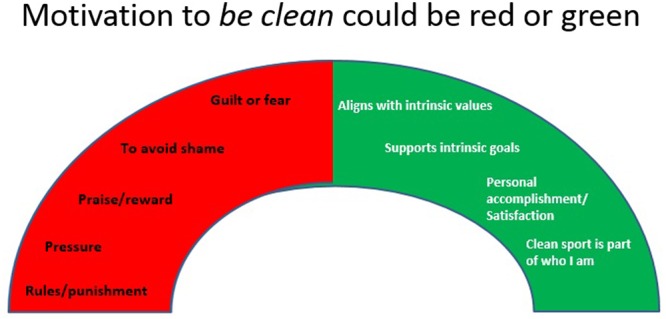
Motivation barometer.

Both experimental workshops use an interactive approach where coaches are encouraged to ask questions and discuss content amongst fellow participants and with the presenters. There are a number of activities included in the design of the experimental workshops which invite participants to actively participate. For instance, coaches are invited to write down on sticky notes what they might perceive to be some of the reasons athletes might want to be ‘clean,’ that is not use prohibited substances. They are then provided with an A3 laminated ‘motivation barometer’ (**Figure 1**) and asked to place the sticky note in a position on the barometer that best suits the identified reasons. Other activities involve discussions of videos, role playing of a hypothetical coach-athlete interaction, and group activities which aim to apply theoretical principles to specific sport situations (e.g., discuss the application of need supportive strategies in situations where a coach is approached by an athlete who wants to use nutritional supplements to speed up their strength recovery from an injury).

#### Additional Content and Resources

Additional resources were developed to complement the workshops in each condition and to improve participant adherence.

##### Control condition

A list of websites is available on the final slide of the workshop and an A4 hard copy of the list is distributed to participants. The list signposts the WADA website, the relevant NADO website, how to check for banned substances in medication, and the process of reducing the risk of inadvertent doping through supplement use. In addition, coaches are provided with information on how they can report any suspicions of doping to the relevant authorities. A hard copy of the slides is also provided to coaches.

##### Experimental condition

**FIGURE 2:**
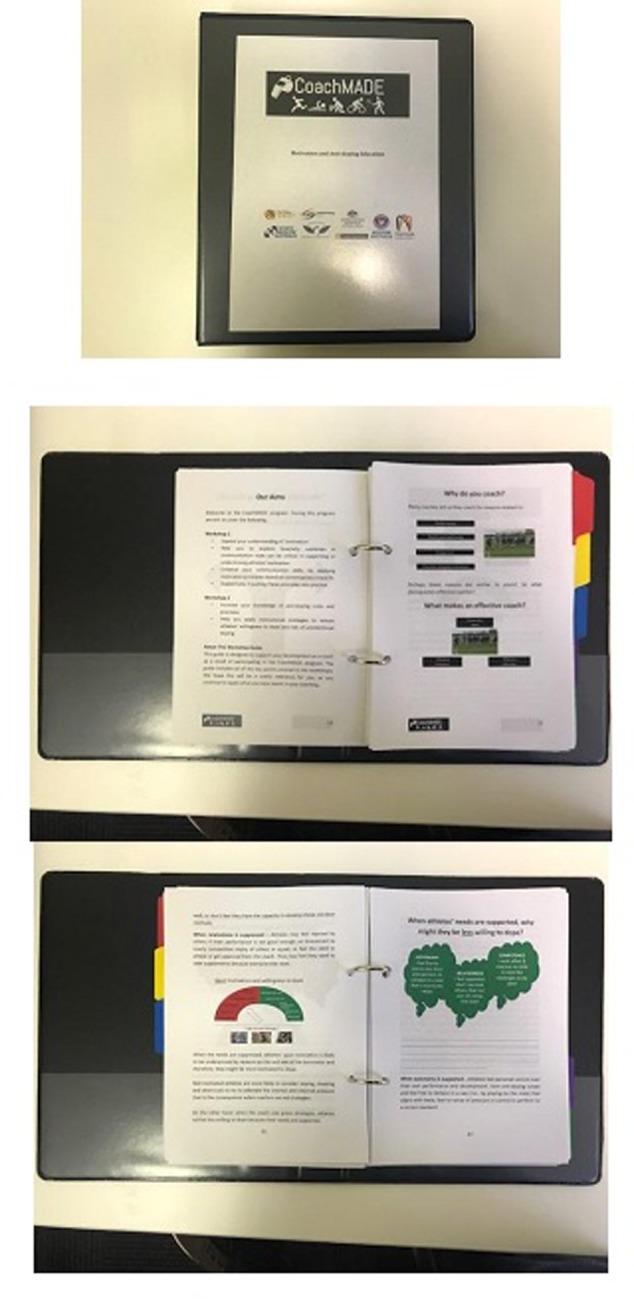
Workbook examples.

The coaches in this condition receive the same information as those in the control condition. In addition, A5 workbooks are provided which include (**Figure 2**):

• Content from the presentation slides with questions to check understanding and summaries of main points.
• Descriptions of the practical activities run in the workshops.
• A personal action plan for coaches to implement the motivational strategies taught in the workshops. We created action and coping planning sheets for coaches to think through how/when strategies might be used, what challenges they might face in doing so, and how these challenges can be overcome.
• Instructions on a week-by-week basis regarding the practicalities of implementing motivational strategies when communicating with athletes. Detailed descriptions are given on how to implement these strategies before, during, or after training. Coaches can keep a reflective diary of success and failures in implementing in their coaching sessions what they have learned at the workshops.

The workbooks are designed to be portable to allow coaches to carry them during training sessions and to take notes. The coaches are encouraged to utilize the workbooks throughout the program.

The project teams have also created restricted access Facebook groups for participating coaches in the experimental condition in each country. The Facebook groups provide coaches with the opportunity to engage in discussions with the project team and interact with fellow coaches participating in the program. For coaches not on Facebook, they receive a weekly email communication from the project team. The team provides weekly prompts via Facebook or email to remind coaches to continue to implement the strategies taught during the workshops, as outlined in their workbooks. In addition, the groups are used to disseminate topical information, including research publications, news articles, and videos illustrating different motivational strategies/communication styles by coaches. We have also created videos with amateur actors depicting different styles of coach communication. These video clips are designed to assist the coaches to grasp the motivational concepts discussed during the workshops. The coach actors in the video clips reinforce to the athlete actors the importance of providing accurate information about the risks of supplement use. However, different video clips have been created (each about 5 min long) in which a coach is interacting with an athlete in need supportive, need thwarting, or motivationally neutral ways.

### Piloting of the Material

Pilot deliveries of the intervention workshops have been conducted across all three countries using between 5 and 7 coaches of varying experience. Following the workshops, coaches were invited to provide feedback via evaluation questionnaires. During these workshops, coaches were able to provide comments on the general workshop approach/delivery in addition to the specific interactive activities and material that we prepared. The feedback has been very positive and will be presented in a future process evaluation publication.

### Phase 2

In this phase, we are currently delivering a cluster randomized control trial in Australia, United Kingdom, and Greece. This study is a parallel group, two-condition, superiority trial. Due to calendar differences in the start of sport seasons, the intervention started half a year earlier in Australia than in the other two countries. Recruitment is ongoing in Greece and the United Kingdom and has been completed in Australia, hence the description of the methodology in this section is written in future tense.

#### Participants and Recruitment

We will recruit from sport clubs 20 full-time or part-time coaches in each condition from each country, giving a total sample size of 120 coaches and estimated 1200 athletes. There are no exclusion criteria for coaches based on their own demographic or coach history characteristics. Inclusion criteria include having a minimum of six athletes who are 14 years or older, train at least once a week, and compete on a regular basis. No more than six coaches will be recruited from any given club, and coaches within that club will be allocated to the same condition. Due to the number of recruited participants, we will use a staggered recruitment design. We will aim to recruit from a variety of sports in all three countries, and from both male and female coach and athlete samples. Power estimates have been calculated with the Optimal Design Software (Spybrook et al., 2011) for clustered RCTs with treatment at level 2, primary outcomes at level 1, estimated average number of athletes per coach to be 10, intraclass correlation coefficient of 0.005, small effect size (δ =0.22) for willing to engage in doping, and oversampling by about 30% to counter possible missing values and coach/athlete dropout from the study. Clubs will be assigned randomly to either a control or intervention condition with a 1:1 allocation using permuted blocks of random sizes. The block sizes will not be disclosed to ensure concealment. A researcher will carry out randomization (and allocate clubs to the two arms) via a computer software following recruitment. Allocation concealment will be ensured, as randomization will not be disclosed until after the intervention starts. All research assistants who will collect data will be blind to condition allocation. There will be no circumstances in which unblinding for those individuals will be necessary. Due to the nature of the intervention, participants cannot be blinded to allocation, but they will be strongly encouraged not to discuss the content of their training with coaches from other sport clubs until after the end of the program.

Recruitment will be carried out by the research team who will liaise with sport governing bodies, coach organizations, and sport clubs in and around Perth (Australia), Thessaloniki (Greece), and Leeds (United Kingdom). Forms of recruitment will include face-to-face contact (e.g., meetings with club president or providing an information session to coaches), flyers, information delivered via email, and promotion via social media (e.g., twitter) with links to the project’s website (e.g., for the Australian website^3^).

### Procedure

**Table 1** demonstrates an overview of the process involved for coach and athlete participation for each condition. In summary, the intervention period starts in week 1 and ends in week 12. Coaches and athletes in each condition provide measures prior to week 1. Then, coaches in the intervention condition will receive the first workshop in week 1 and the second workshop in week 5. Coaches in the control condition receive their workshop in week 1. Coaches in the intervention condition are given weekly tasks each week up to week 12. The weekly tasks in the first 4 weeks focus on planning activities that aim to increase the use of need supportive communication and avoid or minimize the use of need thwarting behaviors when coaches are interacting with their athletes. The weekly tasks in weeks 5–12 focus on planning activities that aim to help coaches to initiate discussions about doping issues using a need supportive communication style. As explained earlier, coaches are assisted in these weekly activities via a number of resources available in their workbooks and via the private Facebook group. Coaches and athletes in both arms provide assessments after the intervention, that is, in week 13, as well as 2 months later. The coaches in the control condition will receive the motivational workshop (and all associated resources) after all assessments have been completed. However, due to resource constraints, they will not have the ongoing support from us should they wish to put in practice what they have learned.

**Table 1:** The SPIRIT schedule of enrolment, interventions, and assessments.

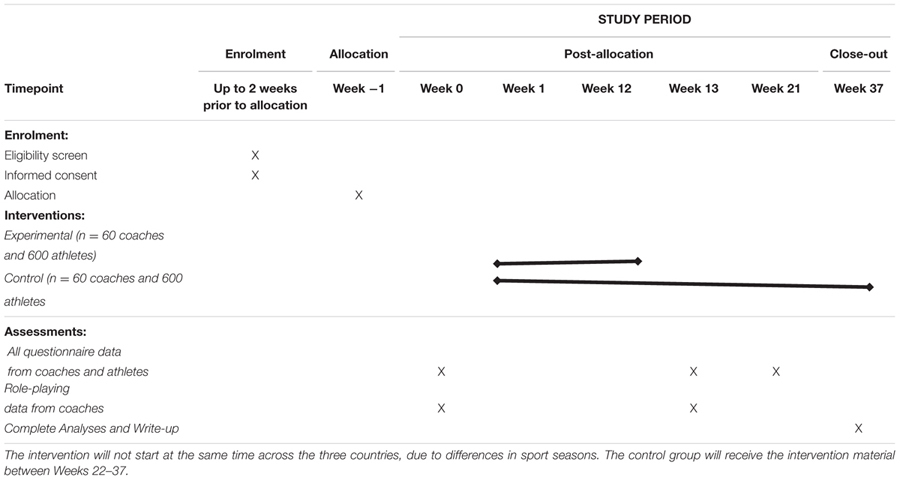

To promote participant retention, athletes will be entered into a prize draw to win monetary retail vouchers if they complete all assessments. In order to promote retention of coaches in the trial, the research team will be in regular contact with them throughout the intervention period via phone and email. There will be no other data collected from athletes or coaches if they refuse to continue participation in the study. All coaches will receive a certificate from the research team and a monetary retail voucher if they complete all aspects of the project.

The workshops will be delivered by our research team members who have expertise in psychology and experience in delivering workshops about motivation or anti-doping to coaches and athletes. To standardize the delivery of the workshops across countries, presenters at the workshops will be trained using video recorded demonstration workshops and detailed slide notes. The workshops will take place in a prearranged central location with access to suitable teaching facilities (i.e., close seating arrangements, projector). These locations include university campuses, local sport and recreation departments, or at participating sporting clubs or organizations.

### Measures

#### Questionnaires

Two questionnaire packs (one for coaches and one for athletes) with the same questions will be administered at all three time-points (with the exception of questions about demographics which will be administered at baseline). Participants will be matched up across time points by a unique ID code given to them by the research team. The questionnaire packs include new questionnaires developed by us for the purposes of this project as well as questionnaires that are already available in the literature; we refer the reader to the original sources for information on the psychometric properties of established questionnaires.

##### Athlete questionnaire pack

(1) Willingness to take potentially prohibited substances (Whitaker et al., 2014). It consists of nine items, scored on a 1 (*not at all willing*) to 7 (*extremely willing*) scale. The stem is “Would you be willing to use a banned substance if you…”: An example item is: “Have been heavily underperforming?”
(2) Moral disengagement in doping (Kavussanu et al., 2016). It consists of six items, scored on a 1 (*strongly disagree*) to 7 (*strongly agree*) scale. An example item is: “Doping is alright because it helps your team.”
(3) Attitudes toward doping (Barkoukis et al., 2013). It consists of eight items, scored on a 1–7 scale with opposite adjectives. The stem is “How do you feel about doping?” An example item is: “Harmful” (scored as 1) and“Beneficial” (7).
(4) Efficacy to resist doping-related temptations (Barkoukis et al., 2013). It consists of six items, scored on a 1 (*no confidence*) to 7 (*complete confidence*) scale. The stem is “How confident would you be that you could resist the temptation to use banned substances even if…?” An example item is: “Your teammates or other competitors were using these substances?”
(5) Self-reported use of performance/recreational substances and drugs. This variable is assessed with a new measure developed by the research team. We list 13 groups of substances and products, some legal (e.g., creatine) and some illegal (e.g., anabolic steroids), and ask athletes whether they have used any of those in the last 12 months (in the first assessment) or since last completing the questionnaire (in the next two assessments).
(6) Knowledge about anti-doping testing procedures. This variable is assessed with a new measure developed by the research team and based on current NADO knowledge assessment approaches. It is presented in the form of a quiz with six questions with three possible answers (*True, False, I Don’t Know*). An example is: “If a nutritional supplement is bought from the pharmacy (over-the-counter), it will not contain a banned substance.”
(7) Behaviors to prevent unintentional/inadvertent doping. This variable is assessed with a new measure developed by the research team. We list six behaviors and we ask athletes to indicate with a *Yes* or *No* answer whether they have engaged in this behavior in the last 4 weeks. An example is: “Checked if my supplements, food and/or drinks contain banned substances.”
(8) Perceived need supportive and need thwarting coach behaviors (Interpersonal Behaviors Questionnaire (IBQ) in Sport; Rocchi et al., 2017). It consists of 24 items, scored on a 1 (*strongly disagree*) to 7 (*strongly agree*) scale. The stem is “Over the last 4 weeks, my coach….” An example item for need support is: “Supported my decisions” and for need thwarting is: “Imposed their opinions on me.”
(9) Satisfaction (Bartholomew et al., 2011a) and frustration of psychological needs (Bartholomew et al., 2011b). Fifteen items measured the satisfaction of the three psychological needs and 12 items measured the frustration of those needs. All items were scored on a 1 (*strongly disagree*) to 7 (*strongly agree*) scale. An example item for need satisfaction is: “I have a say regarding what skills I want to practice” and for need frustration is: “I feel forced to follow training decisions made for me.”

##### Coach questionnaire pack

(1) Moral disengagement (moral disengagement in doping scale, Kavussanu et al., 2016). See athlete questionnaire pack.
(2) Attitudes toward doping (Barkoukis et al., 2013). See athlete questionnaire pack.
(3) Efficacy to discuss with athletes about doping (Doping Confrontation Efficacy Scale, Sullivan et al., 2015). This variable is a 20-item scale, but we are using seven items only as the rest of the questions mix discussions about doping and communication style used in such discussions (e.g., “how confident are you in your ability to confront athletes about PEDs while avoiding personal criticism?”). Unlike Sullivan et al. who used a 1–7 scale, we used a 0% (*no confidence*) to 100% (*complete confidence*) scale, as such a scale is more common in the self-efficacy literature (Bandura, 1997). An example item from those we are using is: “How confident are you in your ability to discuss banned substances and methods with an athlete?”
(4) Perceived effectiveness of need supportive and need thwarting communication styles in dealing with a situation in which a coach suspects an athlete in their team has used a banned substance. This variable is assessed with a new measure developed by the research team. We developed 10 items, five for a need supportive style and five for a need thwarting style; these items are scored on a 1 (*very ineffective*) to 7 (*very effective*) scale. An example item of the former style is: “Demonstrating affection and care,” and of the latter is: “Impose rules with no explanations.”
(5) Efficacy to create anti-doping atmosphere within the team. This variable is assessed with a new measure developed by the research team. We wrote four items which are scored on a 0% (*no confidence*) to 100% (*complete confidence*) scale. An example is: “How confident are you to create a culture within your athletes in which doping is not valued?”
(6) Knowledge about anti-doping testing procedures. See athlete questionnaire pack.
(7) Encouragement of athletes to engage in behaviors to prevent unintentional/inadvertent doping. This variable is similar to the ‘behaviors to prevent unintentional/inadvertent doping’ questionnaire in the athlete pack, but it has been modified to ask coaches whether they have encouraged their athletes to engage in those behaviors.

##### Fidelity assessment

Coaches are also asked to participate in a semi-structured role play. During the role play, a trained research assistant plays the role of an athlete considering taking banned substances while the coach responds in ways which they would consider as a typical response from them. In this hypothetical scenario, a 23-year-old athlete has experienced a performance ‘plateau’ in that they haven’t seen their performance improve over the last 12 months. They are considering taking performance enhancing supplements to break this trend. The role play typically lasts about 20 min and takes place on two occasions, before the intervention and after the end of it, as indicated in **Table 1**. The role plays are audio recorded so that they can be coded later on in terms of the communication style used by the coach. We will use an observational scale (Quested et al., under review) with trained raters, blinded to the experimental condition, to rate the frequency and intensity of need supportive and need-thwarting communication used in these discussions. The aim of this assessment is to establish whether the intervention arm coaches’ communication during the second role play demonstrates fidelity to the intervention (i.e., “treatment enactment”; see Borrelli, 2011), and also to compare the ratings of the coaches in the two arms at each time point. A more rigorous test would have been to code actual communications between coaches and their athletes, however, this was not feasible due to resource constraints as well as the logistical and ethical challenges of filming such discussions. Our role playing is, hence, a proxy measure of fidelity to treatment enactment. We have followed many of Borrelli’s recommendations to enhance fidelity throughout the research process including study design (e.g., explicit use of a theoretical model, pilot testing, and feedback from participants), training (standardized training, accommodate learner differences, assess skill acquisition, and prevent skills drift), treatment delivery (interviews at the end of the project, use of a manual), and treatment receipt (e.g., present material in an engaging way, assess confidence to apply the skills delivered).

### Phase 3

In this last phase of the project, we will implement a thorough process evaluation of the intervention via coach and athlete interviews, coach questionnaires on ease and usefulness of the training material, as well as fidelity to the protocol assessments. We plan to interview either individually or in a group format between 5 and 10 coaches and athletes in each country from the experimental condition. The purpose of the interviews with the coaches will be to ascertain their views on the training they received and its different components (e.g., workshops, online material). We will establish which components the coaches liked or disliked (and why), and how often they engaged with those components in their coaching practice. We will also discuss successes and challenges the coaches experienced in integrating the training material in their coaching practice. Suggestions will also be sought from the coaches in terms of material modification and, more generally, in terms of program modification. This qualitative information will be combined with quantitative ratings that coaches (from both arms) will provide at the end of each workshop and at the end of the study. These ratings will provide evaluations of the perceived usefulness and clarity of the different components of the training and the coaches’ efficacy in utilizing those. The interviews with athletes will establish whether the athletes noticed any changes in their coaches’ communication (in terms of content and style) about (anti)doping from before to after the program.

Limitations of the project include the lack of strong fidelity data as to whether the coaches will implement the taught strategies with their athletes, the use of self-reported data for doping use, and also the fact that the anti-doping education will be delivered by researchers and not by practicing anti-doping educators. Also, other influences on athletes’ tendencies to engage in doping, besides the coach influence, such as societal pressures or organizational pressures at the sport club level are not assessed in this project. Another potential limitation of the project is that the two groups will receive unequal attention in terms of hours of face-to-face contact and online support (related to the implementation of need supportive communication). It would not have been desirable to offer similar amount of time to the control group because there is only so much fuctual information about anti-doping procedures and banned substances one can deliver in an engaging way in face-to-face workshops. Our current 1-h workshop for the control group reflected current anti-doping practice in the three countries. The other option would have been to cut down our motivation intervention to a 1-h workshop and provide nothing more. However, the meta-analysis by Su and Reeve (2011) shows that SDT interventions need to be fairly extensive to be effective. Having a third group (attention control) would have also not been financially feasible given the size of the project. On the other hand, intensive interventions do not necessarily produce more positive results. For example, such interventions can result in participant attrition due to lack of time for engagement from the participants.

### Data Management

All hardcopy data will be stored in locked filing cabinets at the participating universities. A document linking participants’ IDs (necessary to match up coach and athlete data at different time points) with participants’ names will be kept securely on a password-protected computer and stored on University secure servers. All data will be kept securely for the number of years stipulated at each participating university (e.g., 7 years at Curtin University), after which they will be destroyed. Data will be entered into a computer spreadsheet by trained research assistants; a subset of that data will be double-checked by members of the research team for accuracy.

### Analyses

The main statistical analyses of the questionnaire data will involve multilevel modeling, accounting for both the longitudinal and nested (athletes within coaches) nature of the data. Country differences will be tested with contrast effects within multilevel modeling. Data from those who dropout from the study will be included in the analyses using intention-to-treat principles. We will compare the results obtained from these analyses with those obtained from a “per protocol analysis” (i.e., excluding participants who dropped out). In terms of missing data, results will be compared using imputation methods and multilevel modeling methods for handling such data. Analysis of the role-playing videos for fidelity assessments will involve repeated-measures MANOVAs. Interviews will be analyzed thematically, within and across countries, by using deductive and inductive approaches to identify patterns in the data (Braun and Clarke, 2006).

### Ethics and Dissemination

Ethics approval for this project has been granted by Curtin University’s Human Research Ethics Committee (HRE2016-0345); reciprocal ethics approval has been granted by the other two participating universities. Any significant modifications to the project will be submitted for University ethics approval and will be documented subsequently in the Australian and New Zealand Clinical Trials Registry (ANZCTR). To date, there has been one modification to the original protocol, that is, the removal of the age cap (18 years of age) for eligible athletes placed originally. This change was implemented in order to expedite the recruitment process so that it falls within the timeline of the project. The research team makes contact with coaches to determine their eligibility and, if eligible, seeks their written consent. Research assistants (trained by the research team) then distribute, at least 2 weeks prior to the initial data collection, consent/assent forms to athletes. Parental consent forms are also distributed asking parents of athletes between the ages of 14–16 years to complete an opt-out form, if necessary. It is made clear to all participants that they are free to withdraw from the project at any point without providing a reason. In general, the ethical guidelines of the American Psychological Association for research with human participants (section 8^4^) are followed.

At the conclusion of the project, all participating coaches will receive a written summary of the results and practical suggestions for dissemination to their athletes and club administrators. The findings from this study will also be reported to various stakeholder groups via written reports and presentations. These stakeholder groups will include, but may not be limited to sporting clubs or organizations, sport governing bodies, anti-doping organizations, and the International Olympic Committee. The findings will also be disseminated widely via the media offices of participating universities, partner organizations, social media channels including Twitter and the project website, and via peer-reviewed journal articles and conference presentations.

## Conclusion

The project is in line with the revised 2015 WADA Code in which there is increased emphasis on athlete support personnel using their influence to foster athletes’ anti-doping attitudes. The project has the potential to advance the anti-doping literature in several ways. First, this study is the first field intervention aiming to provide anti-doping education to a large group of coaches across three countries using a cluster randomized control trial design. Second, this trial is the first anti-doping education program that incorporates principles of motivational theory in order to help coaches (a) create a need supportive motivational atmosphere within their team (hence, creating an environment which minimizes the temptation for using prohibited substances), and (b) utilize a need supportive communication when discussing with their athletes about doping issues (e.g., testing, checking for medication, implementing preventative behaviors to avoid inadvertent doping). Third, this project will assess longitudinally a variety of outcomes, both at the coach and athlete level, and will implement a thorough process evaluation and dissemination plan. Findings from our project could have implications for the content of anti-doping education programs offered by the World Anti-Doping Agency and national anti-doping agencies.

## Author Contributions

NN, DG, SB, VB, EQ, and LP conceived the project and obtained the project funding. EQ, NN, SB, LW, and LP contributed to the development of the intervention materials. GP, SK, and VB translated all material in Greek. NN has the overall coordination responsibility for the project. BS, LW, and GP/SK manage the project on a daily basis in Australia, the United Kingdom and Greece, respectively. All authors contributed to the refinement of the study protocol and approved the final manuscript.

## Conflict of Interest Statement

The authors declare that the research was conducted in the absence of any commercial or financial relationships that could be construed as a potential conflict of interest.

## Footnotes

1 https://www.asada.gov.au/

2 https://www.galinos.gr/

3 http://www.coachmade.com/

4 http://www.apa.org/ethics/code/index.aspx
